# RETRACTION: Synthesis and Evaluation of PCL/Chitosan/CQD‐Fe Magnetic Nanocomposite for Wound Healing: Emphasis on Gene Expression

**DOI:** 10.1002/elsc.70040

**Published:** 2025-08-13

## Abstract

**RETRACTION:** E.M. Abed, F. Yazdian, A.A. Sepahi, and B. Rasekh, “Synthesis and Evaluation of PCL/Chitosan/CQD‐Fe Magnetic Nanocomposite for Wound Healing: Emphasis on Gene Expression,” *Engineering in Life Sciences* 25, no. 1 (2025): e202400038, https://doi.org/10.1002/elsc.202400038.

The above article, published online on 19 January 2025 in Wiley Online Library (http://onlinelibrary.wiley.com/), has been retracted by agreement between the journal Editor‐in‐Chief, Ralf Takors; and Wiley‐VCH GmbH. Following an investigation by the publisher, the parties have concluded that this article was accepted solely on the basis of a compromised peer review process. Therefore, the article must be retracted.

**RETRACTION:** E.M. Abed, F. Yazdian, A.A. Sepahi, and B. Rasekh, “Synthesis and Evaluation of PCL/Chitosan/CQD‐Fe Magnetic Nanocomposite for Wound Healing: Emphasis on Gene Expression,” *Engineering in Life Sciences* 25, no. 1 (2025): e202400038, https://doi.org/10.1002/elsc.202400038.

The above article, published online on 19 January 2025 in Wiley Online Library (http://onlinelibrary.wiley.com/), has been retracted by agreement between the journal Editor‐in‐Chief, Ralf Takors; and Wiley‐VCH GmbH. Following an investigation by the publisher, the parties have concluded that this article was accepted solely on the basis of a compromised peer review process. Therefore, the article must be retracted.

